# Expansion of mouse castration-resistant intermediate prostate stem cells in vitro

**DOI:** 10.1186/s13287-022-02978-x

**Published:** 2022-07-15

**Authors:** Yalan Xu, Jie Mu, Zhixia Zhou, Yu Leng, Yali Yu, Xiuyue Song, Aihua Liu, Hai Zhu, Jing Li, Dong Wang

**Affiliations:** 1 Institute for Translational Medicine, The Affiliated Hospital of Qingdao University, Medical College, Qingdao University, Qingdao, 266021 China; 2 School of Basic Medicine, Qingdao University, Qingdao, 266021 China; 3 College of Life Sciences, and School of Pharmacy, Medical College, Qingdao University, 308 Ningxia Road, Qingdao, 266071 China; 4 Department of Urology, Qingdao Municipal Hospital Affiliated to Qingdao University, Qingdao, 266011 China

**Keywords:** castration-resistant prostate cancer, Stem cell, Intermediate cell, Androgen receptor, Prostate-specific antigen, Androgen deprivation, Enzalutamide

## Abstract

**Background:**

Most castration-resistant prostate cancers (CRPCs) have a luminal phenotype with high androgen receptor (AR) and prostate-specific antigen (PSA) expression. Currently, it is difficult to culture castration-resistant luminal cells with AR and PSA expression.

**Methods:**

We formulated a custom-made medium and isolated primary cells from the prostate of adult wild-type (WT) and TRAMP mice. The cells were characterized by immunofluorescence staining, transcriptomic analysis, and qRT-PCR verification. Their self-renewal and differentiation potential in vitro and in vivo were examined. We treated the cells with androgen deprivation and enzalutamide and performed immunofluorescence staining and western blotting to analyze their expression of AR and PSA.

**Results:**

We isolated a novel type of castration-resistant intermediate prostate stem cells (CRIPSCs) from adult WT and TRAMP mice. The mouse CRIPSCs proliferated rapidly in two-dimensional (2D) culture dishes and can be cultured for more than six months. The mouse CRIPSCs expressed luminal markers (AR, PSA, and *Dsg4*), basal markers (CK5 and p63), *Psca*, and the intermediate cell marker (*Ivl*). Transcriptomic analysis showed that the mouse CRIPSCs had upregulated signaling pathways related to cancer development and drug resistance. In the long-term culture, TRAMP CRIPSCs had higher expression of the genes related to stem cells and cancers than WT mice. Both WT and TRAMP CRIPSCs formed organoids in Matrigel. WT CRIPSCs did not form prostate tissues when transplanted in vivo without urogenital sinus mesenchyme (UGM) cells. In contrast, TRAMP CRIPSCs formed prostate ducts in NOG mice without UGM  cells and differentiated into luminal, basal, and neuroendocrine cells. Androgens regulated AR translocation between the nucleus and cytoplasm in the mouse CRIPSCs. Treatment of androgen deprivation  (ADT) and enzalutamide reduced AR expression in WT and TRAMP CRIPSCs; however, this treatment promoted PSA expression in TRAMP, while not WT CRIPSCs, similar to the clinical observations of CRPC.

**Conclusions:**

Our study established a method for isolating and expanding mouse CRIPSCs in 2D culture dishes. Mouse CRIPSCs had markers of basal and luminal cells, including AR and PSA, and can differentiate into prostate organoids and tissues. TRAMP CRIPSCs had elevated PSA expression upon ADT and enzalutamide treatment. Our method can be translated into clinical settings for CRPC precision medicine.

## Background

Prostate cancer is the second most cancer type in men globally. It is commonly diagnosed by the level of prostate-specific antigen (PSA), which is regulated by androgen receptor (AR). AR is the major transcription factor regulating prostate development and plays a crucial role in prostate cancer [1–3]. Androgen deprivation therapy (ADT) is the most common first-line treatment for prostate cancer [4]. Tumor mass and AR expression will regress temporarily after ADT treatment; however, most patients will eventually develop castration-resistant prostate cancer (CRPC) and re-acquire high levels of AR and PSA [1, 5]. Thus, the AR signaling pathway is the main target for next-generation therapies [6]. For example, abiraterone and enzalutamide are two new approved drugs targeting androgen synthesis and AR, respectively [5]. They showed some improvement although the patients will inevitably develop drug resistance after a few months [5, 7, 8]. Currently, CRPC is incurable [5].

A central question in CRPC research is to find out the cellular origins of castration resistance. There are adult stem cells in the basal and luminal layers of the prostate [9–13]. By immunohistological studies on animal tissues, it has been well characterized that there are castration-resistant prostate stem cells, which are potential candidates for the cellular origins of CRPC [9]. Upon androgen deprivation by chemical or surgical castration, most prostate luminal epithelial cells will die; however, re-administration of androgens will regenerate the entire prostate tissues again, implying the existence of castration-resistant prostate stem cells [9]. Wang et al. identified a small number (0.7%) of castration-resistant Nkx3.1-expressing cells (CARNs) in the luminal compartment of the mouse prostate [14]. CARNs express luminal markers CK18 and AR and can differentiate into luminal, basal, and neuroendocrine cells when transplanted into immunodeficient mice in combination with urogenital mesenchymal (UGM) cells [14]. Yoo et al. found another type of castration-resistant Bmi1-expressing cells (CARBs) located in the luminal layer [15, 16]. CARBs also express luminal markers CK8, AR, and stem cell marker Sox2 and can differentiate into the three prostate lineages [15, 16]. Kwon et al. identified Sca-1^+^CK8^+^AR^+^ luminal cells from castrated mouse prostate and found that they can differentiate into the luminal and basal cells when transplanted in immunodeficient mice in combination with UGM cells [17]. The pulse-chase method with bromodeoxyuridine has identified quiescent label-retaining luminal stem cells expressing AR in the prostate [18]. To locate castration-resistant stem cells unbiasedly, Zhang et al. adopted a tet-off system to chase label-retaining dormant stem cells during mouse castration [19]. They found the castration-resistant label-retaining stem cells in the luminal layer and expressed luminal markers, including AR, CK8, CK18, Nkx3.1, Pbsn, and the intermediate marker CK19 [19].

In vitro culture models provide valuable tools for prostate cancer research. There have been some methods for culturing prostate basal or luminal stem cells [20, 21]. The development of precision medicine for CRPC requires isolating and expanding patient-specific castration-resistant cells expressing luminal markers, including AR and PSA. There are reports about culturing patient-derived three-dimensional (3D) organoids from prostate tissues. The 3D organoids are composed of luminal and basal cells and are sensitive to androgen deprivation [22–24]. Two-dimensional (2D) culture is convenient for most laboratories. Zhang et al. established a 2D culture system for expanding luminal progenitor cells expressing AR, PSA, and CK18 [20]. The cultured prostate cells can be genetically modified to mimic tumor formation [20, 25]. These cultured luminal cells are still sensitive to ADT in vitro.

This study aimed to establish a method for in vitro expanding prostate luminal stem cells that can resist ADT and express AR and PSA, which will benefit CRPC research. Our previous study established a method to expand adult epithelial progenitor cells from mouse lens epithelium [26]. We found that TGFβ/Smad inhibition was necessary for maintaining adult epithelial identity in vitro. The addition of growth factors (e.g., bFGF) promoted cell proliferation; however, it led to the differentiation and loss of epithelial identity [26]. In addition, ROCK inhibitor Y27632 can promote cell survival [27] and prostate epithelial cell expansion in vitro [20]. The Wnt/β-catenin signaling pathway plays an essential role in prostate development and cancer [28, 29]. Thus, in this study, we formulated a custom-made medium containing TGFβ/Smad inhibitor (A83-01), ROCK inhibitor (Y27632), Wnt/β-catenin pathway agonist (Chir99021), and 10 nM dihydrotestosterone (DHT), without any growth factors. We succeeded in expanding a novel type of castration-resistant intermediate prostate stem cells (CRIPSCs) from wild-type (WT) and TRAMP mice in vitro. They expressed luminal cell markers (AR and PSA), basal cell markers (p63 and CK5), E-cadherin, prostate stem cell antigen (*Psca*), and intermediate cell marker involucrin (*Ivl*). Upon androgen deprivation and enzalutamide treatment, TRAMP CRIPSCs showed reduced AR expression while elevated PSA levels, similar to the clinical observations of CRPC.

## Methods

### Primary cell isolation and culture

The TRAMP mice (Jax#003135) were purchased from The Jackson Laboratories (USA). Genotyping of the TRAMP mice was performed according to the protocol provided by The Jackson Laboratory. WT C57BL/6 J mice were purchased from Ji’nan Pengyue Laboratory Animal Breeding Co., Ltd (China). Primary mouse prostate cells were isolated from the prostate tissues of male WT C57BL/6 J and TRAMP mice of 2–3 months. The cyclic digestion method was used to isolate primary cells. Briefly, mouse prostate tissues, including anterior, ventral, and dorsolateral lobes, were digested in the enzymatic solution containing 2 mg/ml Collagenase I, 2 mg/ml Dispase, 5 mg/ml BSA, and 5 μM Y27632 in DMEM at 37 °C. The cell suspension was collected every 10 min and centrifuged at 1000 rpm for 4 min, and the pellet was re-suspended in PBS. The fresh enzymatic solution was added to the remaining tissues for further treatment until all the tissue pieces were digested. Subsequently, all the cell suspensions were pooled together, centrifuged, and re-suspended in a custom-made prostate cell culture medium containing DMEM/F12 (Invitrogen, Cat# 12,400,024, USA), 2% fetal bovine serum (FBS, Gibco, Cat#10,091,148, USA), 100 U/ml penicillin and 100 μg/ml streptomycin (Gibco, Cat#15,140,122, USA), 5 mM nicotinamide (Sigma, Cat#N0636, USA), 1 mM NAC (Sigma, Cat#A9165, USA), 50 μM Vitamin C (Sigma, Cat#A4403, USA), 3 μM glutathione (Sigma, Cat#G6013, USA), 10 μg/ml insulin (Aladdin, Cat#I113907, China), 7.5 μg/ml transferrin (Sigma, Cat#T0665, USA), 40 nM Sodium Selenite (Sigma, Cat#S9133, USA), 1 μM PGE2, 2 μM Chir99021 (Sellect, Cat#S2924, China), 0.2 μM A83-01 (Tocris, Cat#2939, UK), and 5 μM Y27632 (Sellect, Cat#S1049, China).

Before cell seeding, the culture dishes were incubated with 0.1 mg/ml Matrigel solution diluted in DMEM for one hour and washed with PBS. Accutase (Gibco, Cat#A1110501, USA) was used for cell passaging. During the early four or five passages, the primary cells grew slowly and were passaged at a ratio of 1:1. Many cells died during this period. After P5, i.e., about two months of culture in vitro, we obtained a relatively uniform population of epithelial cells with a high proliferation rate. The split ratio can be 1:20 or more during passaging. 2 × 10^4^ cells can grow to over 10^6^ in a week, with a doubling time of about one day.

### Histology and immunostaining

The cultured cells were fixed in 4% paraformaldehyde (PFA) for 20 min, washed with PBS, permeabilized in 0.1% Triton X-100 for 10 min, and blocked with 5% normal donkey serum for 1 h. Primary antibodies diluted in 5% normal donkey serum and 0.1% Triton X-100 in PBS were added and incubated at 4 °C overnight. Primary antibodies used in this study included: anti-E-cadherin antibody (1:200, 20874-1-AP, Proteintech, China), anti-CK5 antibody (1:100, D220236, Sangon Biotech, China), anti-CK8 antibody (1:100, D220230, Sangon Biotech, China), anti-CK18 antibody (1:200, 66187-1-Ig, Proteintech, China), anti-SOX2 antibody (1:100, 11064-1-AP, Proteintech, China), anti-AR antibody (1:500, ab108341, Abcam, USA), anti-PSA antibody (1:100, 10679-1-AP, Proteintech, China), anti-p63 antibody (1:100, D125020, Sangon Biotech, China), anti-SYP antibody (1:50, 60191-1-Ig, Proteintech, China), anti-Chromogranin-A antibody (1:5000, 60135-2-Ig, Proteintech, China). Secondary antibodies labeled with Alexa Fluor 488, 546, and 647 were purchased from Invitrogen (USA). After 2 h of secondary antibody incubation at room temperature, the cells were washed with PBS at least three times and subsequently stained with 5 µg/ml DAPI (Sigma-Aldrich, USA) for 30 min. Finally, the cells were washed once with PBS, ready for confocal microscopy. Organoids and the cryosections of Matrigel plugs were immunostained by similar protocols as above. Confocal microscopy was performed on a Leica SP8 confocal microscope.

### Organoid formation assay

The cells were digested by accutase, centrifuged at 1200 rpm for 4 min, and mixed in Matrigel at a density of 5 × 10^3^ cells per 100 µl of Matrigel in 24-well plates. The plates were then incubated in a 37 °C incubator for 30 min and added with 500 μl culture medium.

### Cell transplantation in NOG mice

Male NOD.Cg-*Prkdc*^*scid*^*Il2rg*^*tm1Sug*^/JicCrl (NOG) mice of 4–5 weeks were purchased from Beijing Vital River Laboratory Animal Technology Co., Ltd (China). The cells were dissociated with accutase, centrifuged at 1200 rpm for 4 min, mixed with Matrigel at a density of 1 × 10^6^ cells per 200 µl Matrigel, and then injected into NOG mice subcutaneously. The Matrigel plugs were harvested after eight weeks for cryosection and immunostaining.

### Transcriptomic sequencing and analysis

The cells were harvested in Trizol (Invitrogen, Cat#15596026, USA) and stored at − 80 °C. The mRNA library preparation and sequencing were performed on the DNBSEQ-T7 platform of Tsingke Biotechnology (China). The pair-end 150 base reads data were filtered with SOAPnuke (v1.5.2), mapped to the reference genome using HISAT2 (v2.0.4) and Bowtie2 (v2.2.5), and the expression levels of genes were calculated by StringTie (v2.1.2). KEGG pathway analysis was performed using the DAVID Bioinformatics Resources tools (https://david.ncifcrf.gov). The heatmap and bubble plots were drawn by SRplot (http://www.bioinformatics.com.cn) according to the gene expression in different samples.

### Quantitative RT-PCR analysis

Total RNA was extracted in Trizol, and cDNA was prepared by an Evo M-MLV RT Mix Kit (Accurate Biotechnology, Cat#AG11728, China) according to the manufacturer's instructions. The experiment was carried out using the forward and reverse primers listed below, and levels of expression were normalized against GAPDH. The primer list:

*Psca* (Forward: 5′-GGACCAGCACAGTTGCTTTAC-3′ and Reverse: 5′-GTAGTTCTCCGAGTCATCCTCA-3′); *Atg9b* (Forward: 5′-ATGTACCCGAAGGACTCCG-3′ and Reverse: 5′-CATTCCGCTGATGATAGCTGT-3′); *Aldh3a1* (Forward: 5′-TGGCAAAGACTCGTCAGACC-3′ and Reverse: 5′-AGTTCCAAGCACCTATGACAAG-3′); *Krt13* (Forward: 5′- TCCAGAGCGGGACTACAGTG-3′ and Reverse: 5′-ATGATCCGGTTGTTGTCCGTG-3′); *Ly6d* (Forward: 5′-GGTTCCGAGGTCACACAATG-3′ and Reverse: 5′-CAGAGAGCCATAACAGTGAGC-3′); *Ivl* (Forward: 5′-ATGTCCCATCAACACACACTG-3′ and Reverse: 5′-TGGAGTTGGTTGCTTTGCTTG-3′); *lIl33* (Forward: 5′- TCCAACTCCAAGATTTCCCCG-3′ and Reverse: 5′-CATGCAGTAGACATGGCAGAA-3′); *Dsg3* (Forward: 5′- GAAGGTACAAACGTGAATGGGT-3′ and Reverse: 5′-ACTCCAGAAATGCGGTAGGTA-3′); *Col17a1* (Forward: 5′-CAGTTACAGGAGAACGCACTC-3′ and Reverse: 5′-GGCGGGTCATGTGAGCTTT-3′).

### Androgen deprivation and enzalutamide treatment

The cells were cultured and passaged in the media containing different concentrations of DHT and enzalutamide, including the following groups: 10 nM DHT, 1 nM DHT, 0.1 nM DHT, 0 nM DHT, 0.1 nM DHT with 1 μM enzalutamide, 0.1 nM DHT with 10 μM enzalutamide. The total treatment time was about one month.

### Western blotting

The cells were washed with ice-cold PBS three times and lysed with RIPA lysis buffer (Sangon Biotech, China) with a protease inhibitors cocktail (Roche, China). The total protein content in the supernatant was measured by the BCA protein assay kit (Sangon Biotech, China) according to the manufacturer’s instruction. The samples were boiled with SDS-PAGE loading buffer (Solarbio, China). Twenty micrograms of protein was added to each lane, separated in 10% SDS-PAGE, and transferred to polyvinylidene fluoride (PVDF) membranes (Millipore, MA, USA) in a Trans-Blot apparatus (Tanon, China). 5% BSA diluted in TBS-T (0.05% Tween 20 in TBS) was used to block the membranes, and the primary antibodies with optimized concentrations, including AR (1:1000, ab108341, Abcam, USA), Actin (1:1000, D191048, Sangon Biotech, China), PSA (1:1000, 10679, Proteintech, China), were added to the membranes overnight at 4 °C. The membranes were incubated with the corresponding secondary antibodies conjugated with HRP (1:10000, D110011, Sangon Biotech, China) for 1 h at room temperature, and the bands were visualized by an enhanced chemiluminescence detection system.

### Statistical analysis

All statistical analyses were conducted using GraphPad Prism 8 software. Data are presented as mean ± standard deviation. The significant differences were analyzed using ANOVA followed by Bonferroni post hoc tests. Statistical significance was defined as *p < 0.05, **p < 0.01, ***p < 0.001.

## Results

### Expand adult mouse CRIPSCs on 2D culture dishes in vitro

**Fig. 1:**
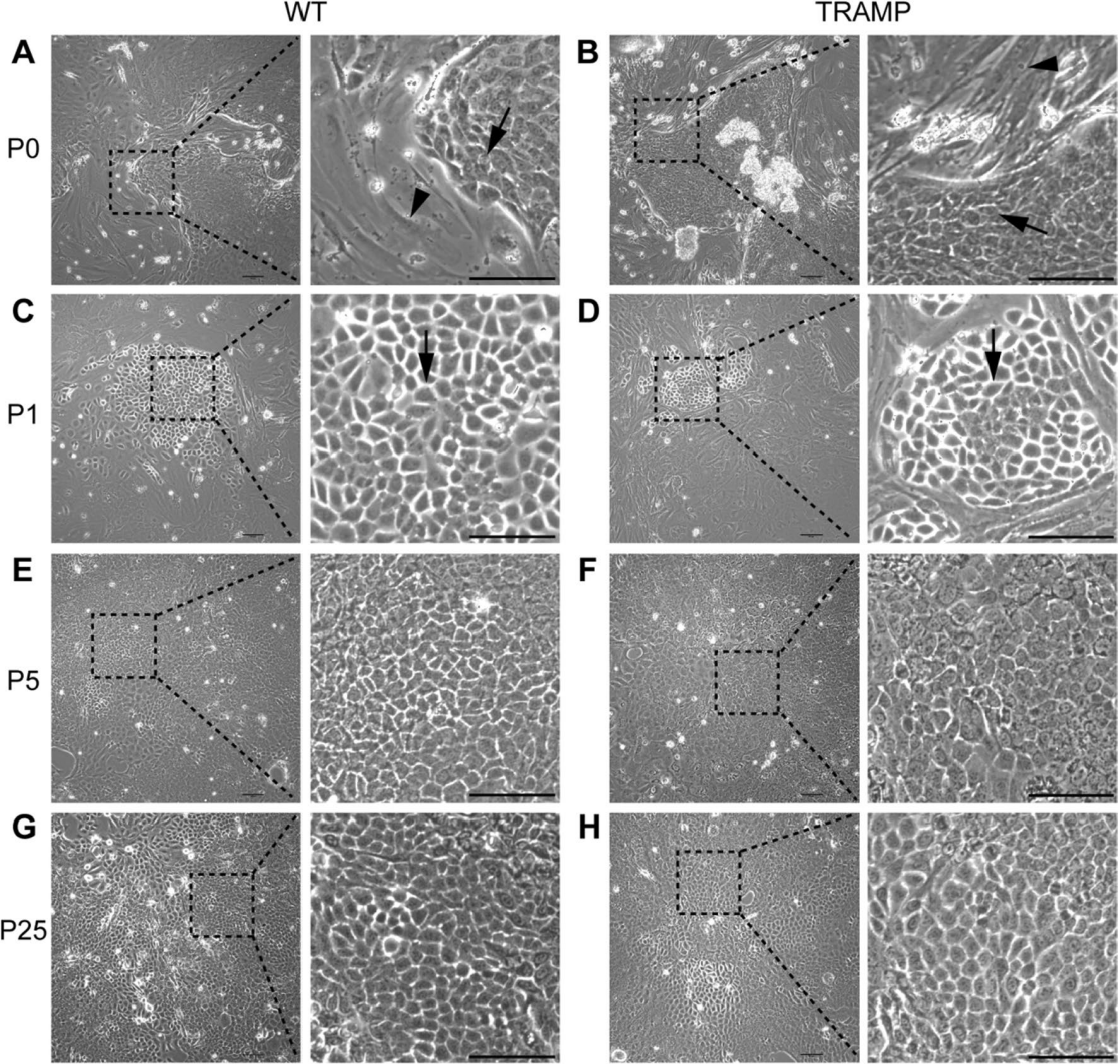
Expansion of CRIPSCs from adult mice. Phase-contrast images of primary mouse prostate epithelial cells isolated from wild-type (WT) and TRAMP mice at primary passage (P0, 7 days, **A **and **B**), P1 (14 days) (**C** and **D**), P5 (2 months) (**E** and **F**), and P25 (6 months) (**G** and **H**). Arrows point to small epithelial cells. Arrowheads point to mesenchymal cells. Scale bars, 100 μm

We isolated primary cells from the prostate tissue of adult WT and TRAMP mice by enzymatic digestion. We used a cyclic digestion method and harvested cell suspension every 10 min from the enzymatic solution. The primary cells contained single cells and small pieces of prostate tissues. The cells would migrate out of the small tissue pieces and populate the culture dishes. The initial passage (P0) had a mixture of cells with epithelial and mesenchymal morphologies (Fig. 1A and B). After the first passage, we noticed some islands of small epithelial cells (Fig. 1C and D). During the early four or five passages, there were many cells dying, and the overall cell proliferation was slow; thus, we passaged cells at a split ratio of 1:1 in this period. The small epithelial cells eventually dominated the population after four or five passages and proliferated rapidly with a doubling time of about one day in the following culture. During each passage, we froze most of the cells and only left 1/20 or less in a new culture dish. The freeze–thaw procedure did not affect the cell morphology, viability, or proliferation. These small epithelial cells referred to as CRIPSCs (Castration-Resistant Intermediate Prostate Stem Cells), can be cultured for more than six months in vitro, with a stable cell morphology (Fig. 1E–H). There was no apparent difference between WT and TRAMP CRIPSCs in the proliferation and morphology (Fig. 1).

### Characterization of the markers of mouse CRIPSCs

**Fig. 2:**
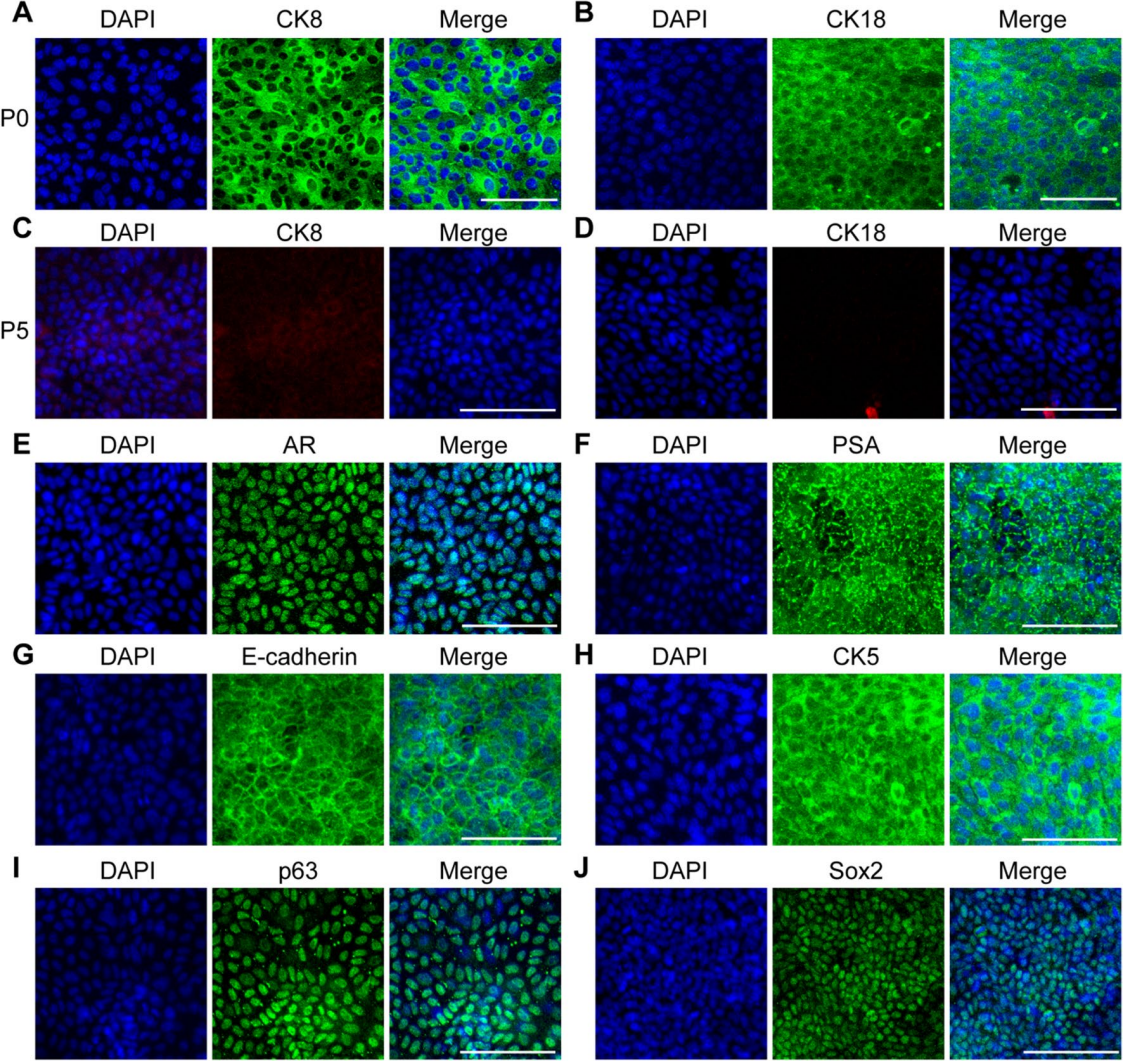
Marker expression of mouse CRIPSCs. The prostate epithelial cells isolated from WT mice at P0 (7 days) (**A** and **B**) and P5 (2 months) (**C**–**J**) were immunostained by the antibodies against CK8 (**A** and **C**), CK18 (**B** and **D**), AR (**E**), PSA (**F**), E-cadherin (**G**), CK5 (**H**), p63 (**I**), and Sox2 (**J**). DAPI-stained nuclei. Scale bars, 100 μm

We first examined the luminal and basal cell markers of mouse CRIPSCs. In the primary (P0) cell culture, the epithelial cells expressed luminal markers CK8 and CK18, which disappeared after about five passages (P5) (Fig. 2A–D). The expanded mouse CRIPSCs of P5 expressed the epithelial marker E-cadherin, luminal markers AR and PSA, and basal markers CK5 and p63 (Fig. 2E–I). Sox2 was temporarily expressed in the cells of early passages; however, barely detected in the cells after P5 (Fig. 2J).

**Fig. 3:**
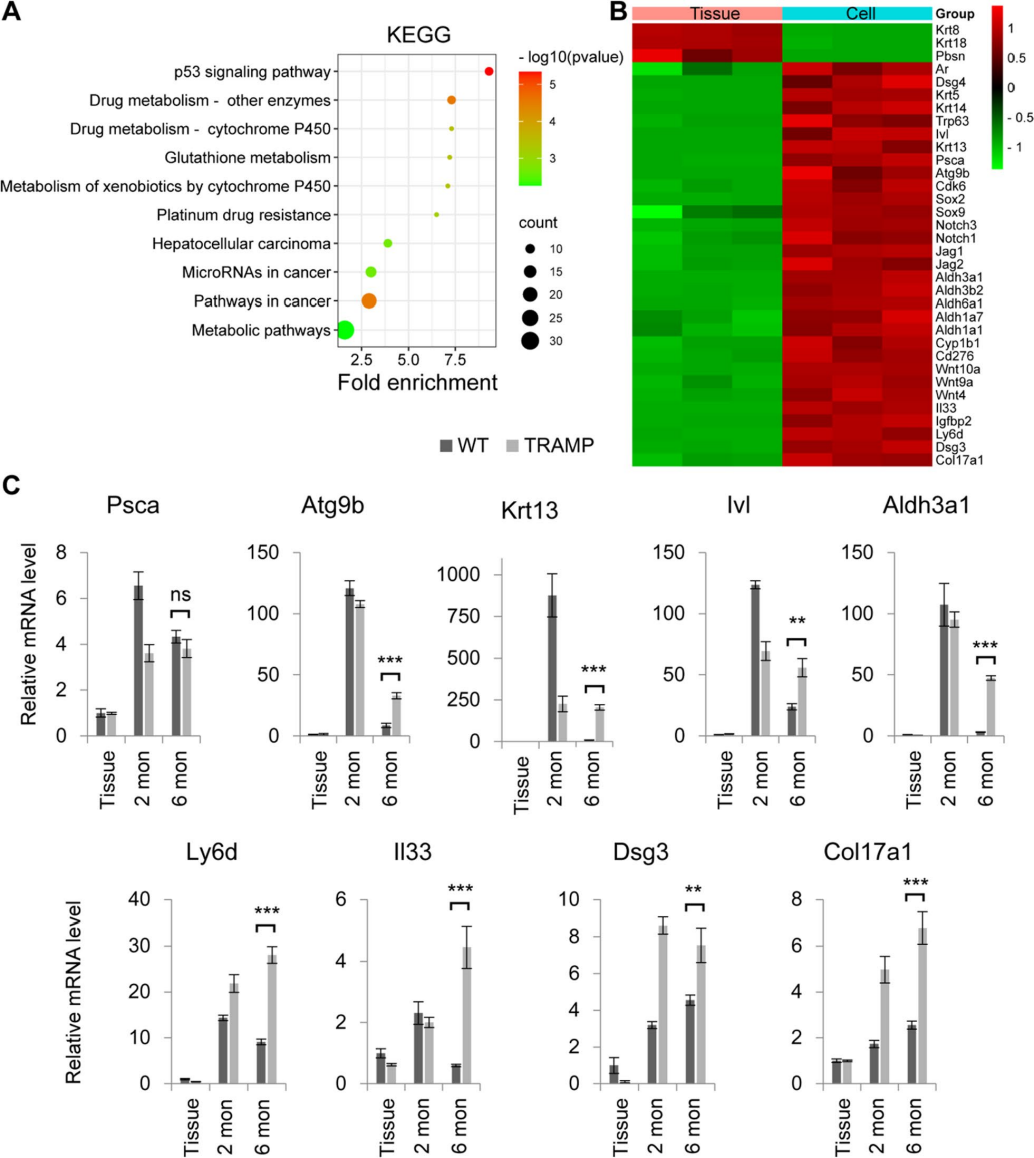
Gene expression profiles of mouse CRIPSCs. **A** KEGG pathway analysis. **B** Heatmap of the differentially expressed genes in prostate tissue and WT CRIPSCs. **C** qRT-PCR analysis of the expression of some genes in prostate tissue, the CRIPSCs cultured for 2 (P5) and 6 (P25) months in vitro, from WT and TRAMP mice. Data were presented as mean ± SD. Two-way ANOVA was performed on the data, followed by Bonferroni post hoc tests. “ns” = not significant. **p < 0.01. ***p < 0.001

We performed transcriptomic sequencing on the prostate tissues and P5 WT CRIPSCs and found that the expanded WT CRIPSCs had upregulated signaling pathways related to cancer development and drug resistance (Fig. 3A). They lost *CK8* (*Krt8*), *CK18* (*Krt18*) and *Pbsn*; however, they still maintained other luminal cell markers, including *Ar* and *Dsg4* [30], together with basal cell markers *CK5* (*Krt5*), *CK14* (*Krt14*), *p63*, and intermediate cell marker *Ivl* (Fig. 3B). WT mouse CRIPSCs also had higher expression of the genes related to stem cells (*Psca, Atg9b, Sox2, Sox9*), cell cycle (*Cdk6*), mammary luminal progenitor cells (*Notch3* and *Notch1*) [31, 32], and cancers (Fig. 3B).

To further examine whether mouse CRIPSCs’ gene expression profiles will change or not during the long-term culture in vitro and the differences between WT and TRAMP cells, we performed qRT-PCR analysis. In the long-term culture for about six months, P25 WT and TRAMP CRIPSCs both expressed a high level of *Psca* (Fig. 3C). TRAMP CRIPSCs had a much higher expression of *Atg9b*, an autophagy gene responsible for stem cell maintenance [33], *Ivl*, a marker for intermediate prostate cells [34], and *Krt13,* a marker of prostate stem cells [35]. P25 TRAMP CRIPSCs also had upregulated genes related to cancer development and drug resistance, such as *Aldh3a1, Ly6d, Il33, Dsg3, and Col17a1* (Fig. 3C).

### Differentiation of mouse CRIPSCs

**Fig. 4:**
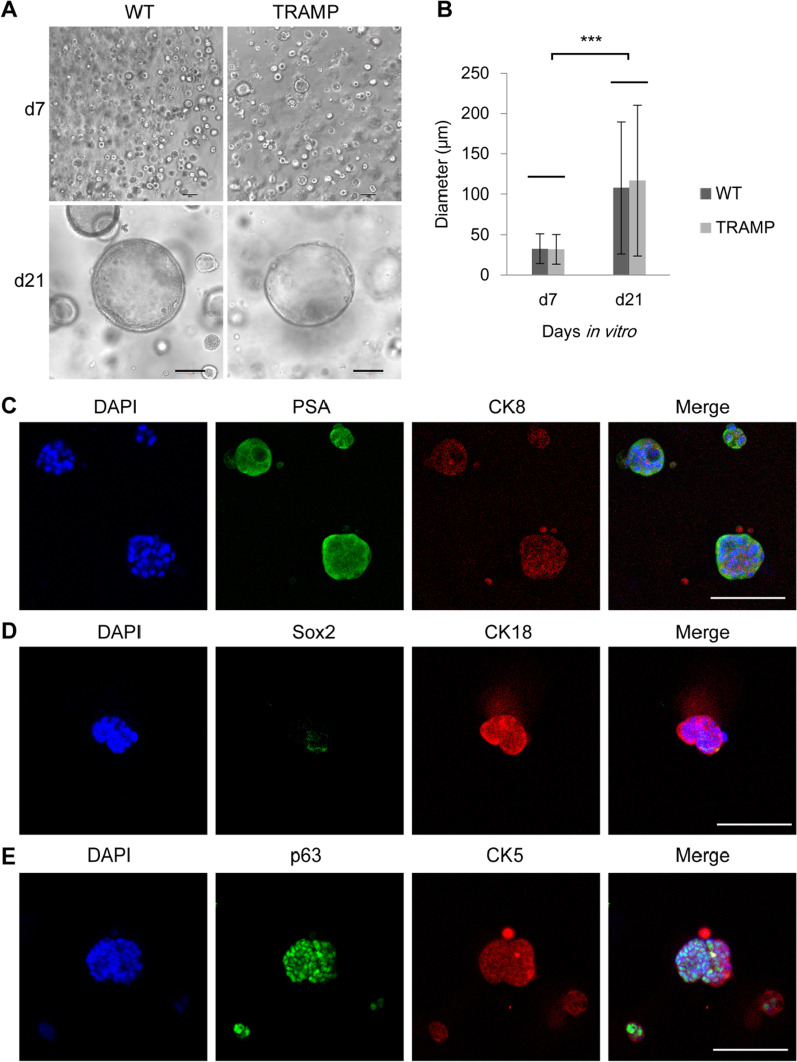
Organoid formation in vitro. **A** Phase-contrast images of the organoids formed by the CRIPSCs (P25, 6 months) derived from WT and TRAMP mice. **B** Quantification of organoid diameter. Data were presented as mean ± SD. Two-way ANOVA was performed on the data, followed by Bonferroni post hoc tests. ***p < 0.001. **C**–**E**, Immunofluorescence images of the organoids stained by the antibodies against CK8, CK18, PSA, p63, CK5, and Sox2. DAPI-stained nuclei. Scale bars, 100 μm

To test the differentiation potential, we mixed P25 WT and TRAMP CRIPSCs within Matrigel and found that they formed 3D organoids and grew into spheres with diameters of hundreds of micrometers during three weeks of culture (Fig. 4A and B). Immunofluorescence staining showed that the organoids expressed luminal markers: PSA, CK8, and CK18; basal cell markers: CK5 and p63; and lost Sox2 expression (Fig. 4C–E).

**Fig. 5:**
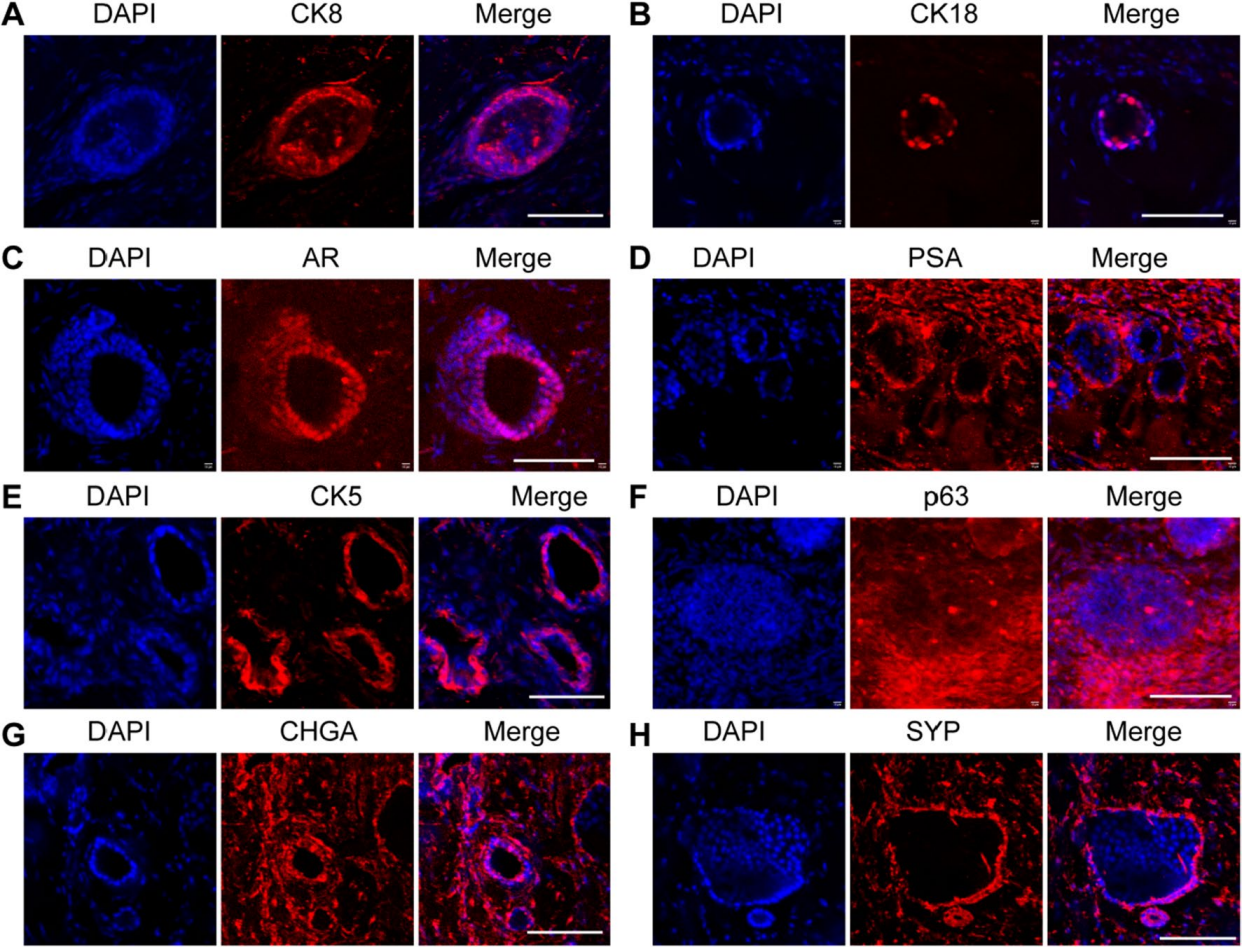
Differentiation of mouse CRIPSCs in vivo. The CRIPSCs (P25, 6 months) isolated from TRAMP mice were transplanted into NOG mice for eight weeks, followed by cryosection and immunostaining (**A**–**H**). The antibodies included CK8 (**A**), CK18 (**B**), AR (**C**), PSA (**D**), CK5 (**E**), p63 (**F**), Chromogranin A (CHGA) (**G**), Synaptophysin (SYP) (**H**). DAPI-stained nuclei. Scale bars, 100 μm

To further investigate the differentiation potential in vivo, we injected WT and TRAMP CRIPSCs mixed in Matrigel into NOG mice subcutaneously. After eight weeks, the Matrigel plugs were harvested for cryosection and immunostaining. We found that only TRAMP CRIPSCs formed prostate ductal tissues in vivo and differentiated into prostate luminal epithelial cells (expressing CK8, CK18, AR, and PSA), basal cells (expressing CK5 and p63), and neuroendocrine cells (expressing CHGA and SYP) (Fig. 5).

### AR and PSA expression of CRIPSCs regulated by androgen deprivation and enzalutamide treatment

**Fig. 6:**
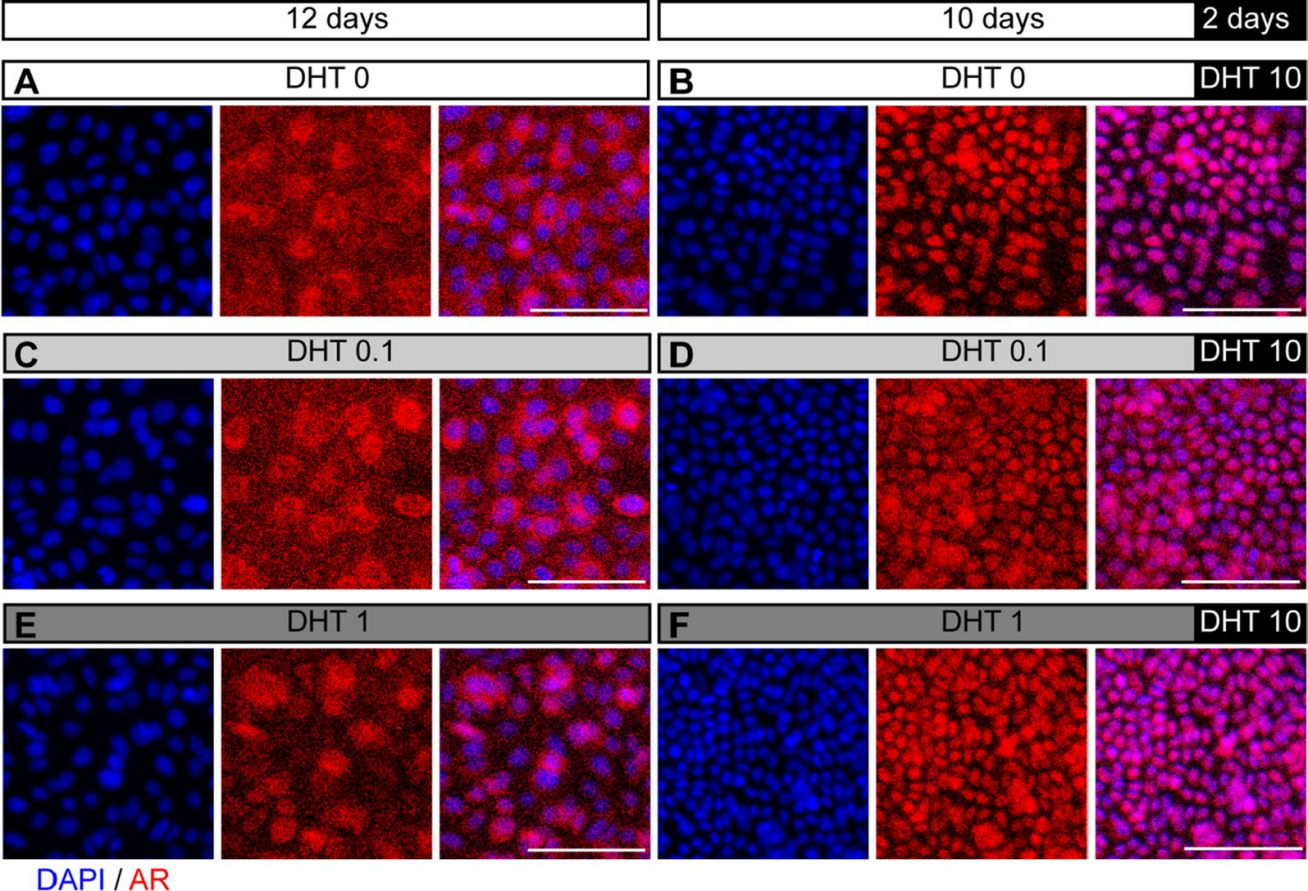
AR translocation between the nucleus and cytoplasm. WT CRIPSCs (P25, 6 months) were cultured in the media supplemented with different concentrations of DHT for 12 days (A, 0 nM; C, 0.1 nM; E, 1 nM) or 10 days of low DHT followed by two days of 10 nM DHT (**B**, **D**, and **F**). DAPI-stained nuclei. Scale bars, 100 μm

AR signaling pathway plays an essential role in prostate development and cancer. AR can be translocated from the cytoplasm to the nucleus upon binding to androgens. Our study expanded mouse CRIPSCs in the medium containing enough amounts of androgen, 10 nM DHT, which made AR located in the nucleus (Fig. 2E). To examine the reversibility of AR translocation, we cultured mouse CRIPSCs in the media supplemented with low concentrations (0, 0.1, and 1 nM) of DHT for 12 days and found that AR was located in the cytoplasm (Fig. 6A, C, and E). The addition of high (10 nM) DHT to the media in the last two days of culture resulted in AR translocation from the cytoplasm to the nucleus (Fig. 6B, D, and F). WT and TRAMP CRIPSCs had similar AR dynamics regulated by DHT.

**Fig. 7:**
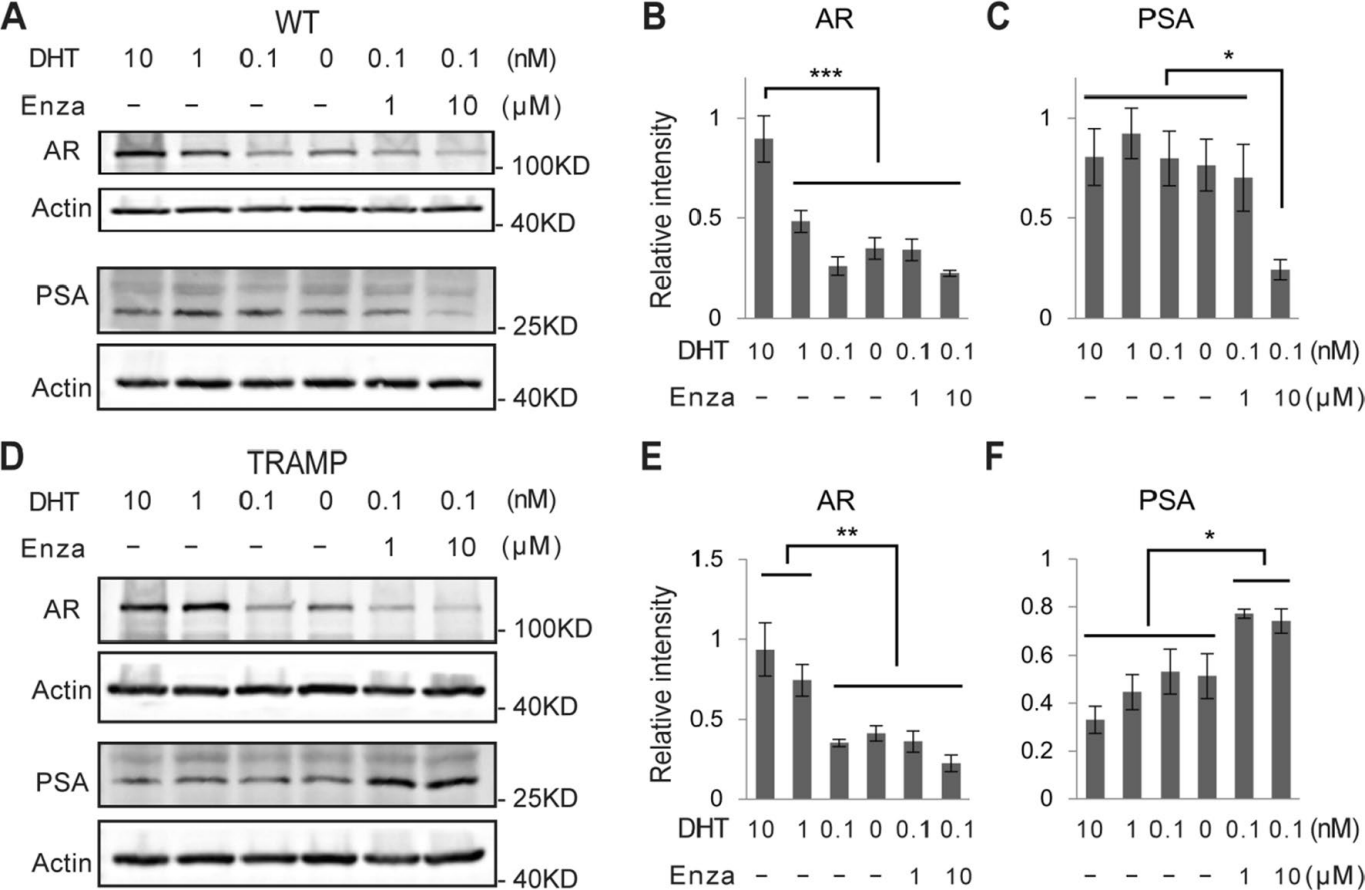
AR and PSA regulation by androgen deprivation and enzalutamide treatment. Western blots and quantifications of AR and PSA of WT (**A**–**C**) and TRAMP (**D**–**F**) CRIPSCs (P25, 6 months) treated with different concentrations of DHT and enzalutamide (Enza) for one month. Data were presented as mean ± SD. One-way ANOVA was performed on the data, followed by Bonferroni post hoc tests. *p < 0.05. **p < 0.01. ***p < 0.001

To mimic the clinical treatments for prostate cancer (ADT and enzalutamide), we cultured P25 mouse CRIPSCs in the media supplemented with different concentrations of DHT and enzalutamide for a month. The proliferation and cell morphology of WT and TRAMP CRIPSCs were not affected by androgen deprivation or enzalutamide treatment. The cells in all these different groups proliferated rapidly and were passaged every week. During each passage, most cells were frozen and only 1/20 or less cells were seeded to a new culture dish. By Western blotting, we found that androgen deprivation reduced the expression of AR in WT and TRAMP CRIPSCs. However, enzalutamide treatment promoted PSA expression in TRAMP CRIPSCs, while not in WT mice (Fig. 7). This result is similar to the clinical observations of CRPC recurrence.

## Discussion

Immunohistological studies and single-cell sequencing analysis show that most CRPCs have a luminal cell phenotype with high AR and PSA expression [1, 36]. Accumulating evidence proved the existence of castration-resistant luminal stem cells [14, 15, 17, 19]. However, expanding castration-resistant luminal cells with AR and PSA expression is challenging. Our study expanded CRIPSCs from WT and TRAMP mice, which expressed the epithelial marker E-cadherin, luminal markers (AR, PSA, and *Dsg4*), basal markers (CK5 and p63), and intermediate cell marker *Ivl*, implying they are an intermediate cell type. The CRIPSCs also expressed prostate stem cell antigen (*Psca*) and cyclin-dependent kinase 6 (*Cdk6*); the latter can associate with AR and enhance its transcriptional activity in prostate cancer cells [37]. This marker expression pattern is similar to the intermediate prostate epithelial cells. During embryonic development, there are many intermediate prostate epithelial cells co-expressing luminal (CK8) and basal (CK5) markers [38]. During postnatal development, the number of intermediate cells decreased [38], and there are still a small number (0.59%) of intermediate cells in the basal compartment of adult prostate tissue of mouse, rat, and human [39, 40]. However, there are few reports about culturing the intermediate cells in vitro.

In our study, the CRIPSCs can be cultured for more than six months with stable cell morphology. There were no apparent differences between WT and TRAMP CRIPSCs in proliferation and morphology after about P5. At the beginning of primary cell culture, there were mixtures of different cell types, including epithelial and mesenchymal cells. There were many cells dying, and the overall cell growth was slow compared to those of late passages. We noticed a transient expression of Sox2 in CRIPSCs at the early passages. Considering the critical roles of Sox2 in stem cells and prostate cancer development [41–43], we think Sox2 may contribute to establishing the stem cell colony at the first step.

Although there was no noticeable difference in the proliferation and morphology between WT and TRAMP CRIPSCs, the latter showed significantly higher expression of the genes related to cancer development and drug resistance (Fig. 3C). For example, *Atg9b* plays an essential role in stem cell maintenance and cancer development under the regulation of the Wnt/β-catenin signaling pathway [33]. In this study, the addition of Wnt/β-catenin signaling pathway agonist Chir99021 may be the reason for *Atg9b* upregulation. Wnt/β-catenin signaling pathway can also activate the intermediate prostate epithelial cell marker *Ivl* [34, 44]. Krt13 is a marker of prostate stem cells [35]. Aldehyde dehydrogenase (ALDH) family members, especially *Aldh1* and *Aldh3a1*, are not only the markers of normal stem cells but also cancer stem cells and play essential roles in maintaining stem cell functions, including proliferation and resistance to cytotoxic drugs in some cancer types, including prostate cancer [45–47]. A recent single-cell sequencing study pointed out that *Ly6d* was a marker for castration-resistant prostate luminal cells and cancer development [48]. *Il33* is related to cancer development, metastasis, and drug resistance [49–52]. *Dsg3* plays an oncogenic role in head neck cancer [53]. *Col17a1* plays an essential role in many malignancies by contributing to cancer cell proliferation and invasion [54].

During prostate development, epithelial–mesenchymal interaction is crucial for proper prostate tissue morphogenesis. Urogenital sinus mesenchyme (UGM) plays a determinant role in prostate tissue formation and can induce other epithelial cells, including bladder, vaginal, mammary, and skin epithelial cells, to form prostate tissue [38]. To examine the in vivo differentiation potential, researchers usually combined prostate stem cells with UGM cells before transplanting them into immunodeficient mice [38]. However, the mechanism underlying UGM induction of prostate tissue formation is not well understood. In this study, we transplanted CRIPSCs into NOG mice without UGM cells and found that WT CRIPSCs did not form prostate ducts, consistent with previous reports; on the contrary, TRAMP CRIPSCs formed well-developed prostate ductal structures with luminal, basal, and neuroendocrine cells. The genes related to stem cells, intermediate prostate cells, and cancer enriched in TRAMP CRIPSCs may contribute to their differentiation into prostate tissue without UGM induction. Further study is needed to investigate the mechanisms underlying the self-induction of TRAMP CRIPSCs.

AR is a core signaling pathway during prostate development, cancer formation, and drug resistance [1]. A cell culture model with AR and PSA expression and castration resistance will be valuable for the research of CRPC. Our immunostaining showed that androgen reversibly regulated AR translocation between the nucleus and cytoplasm (Fig. 6). We performed ADT and enzalutamide treatment on the cultured CRIPSCs for a month to mimic clinical therapies. These treatments downregulated AR expression in both WT and TRAMP CRIPSCs. However, TRAMP CRIPSCs acquired higher PSA expression upon enzalutamide treatment (Fig. 7). This result mimics the high PSA levels during the recurrence of CRPC to some extent.

## Conclusions

Our study established a method for isolating and expanding mouse CRIPSCs on 2D culture dishes. Mouse CRIPSCs had markers of luminal and basal cells. TRAMP CRIPSCs can differentiate into prostate ductal tissues in vivo without recombination with UGM. Under enzalutamide treatment, TRAMP CRIPSCs acquired higher expression of PSA, similar to the situation of CRPC recurrence. Our method can be applied to human prostate cancer tissues for isolating and expanding patient-specific CRIPSCs, which will promote the development of precision medicine.

## Data Availability

The RNA-seq data reported in this paper have been deposited in the Genome Sequence Archive (GSA accession number: CRA007192) and are accessible at the URL: https://ngdc.cncb.ac.cn/gsa/browse/CRA007192.

## Abbreviations

CRPC: Castration-resistant prostate cancers
CRIPSCs: Castration-resistant intermediate prostate stem cells
AR: Androgen receptor
PSA: Prostate-specific antigen
Psca: Prostate stem cell antigen
Ivl: Involucrin
UGM: Urogenital sinus mesenchyme
WT: Wild-type
ADT: Androgen deprivation therapy
CARN: Castration-resistant Nkx3.1-expressing cell
CARB: Castration-resistant Bmi1-expressing cell
DHT: Dihydrotestosterone
