# Correction: Expansion of mouse castration-resistant intermediate prostate stem cells in vitro

**DOI:** 10.1186/s13287-022-03207-1

**Published:** 2022-11-25

**Authors:** Yalan Xu, Jie Mu, Zhixia Zhou, Yu Leng, Yali Yu, Xiuyue Song, Aihua Liu, Hai Zhu, Jing Li, Dong Wang

**Affiliations:** 1 Institute for Translational Medicine, The Affiliated Hospital of Qingdao University, Medical College, Qingdao University, Qingdao, 266021 China; 2 School of Basic Medicine, Qingdao University, Qingdao, 266021 China; 3 College of Life Sciences, and School of Pharmacy, Medical College, Qingdao University, 308 Ningxia Road, Qingdao, 266071 China; 4 Department of Urology, Qingdao Municipal Hospital Affiliated to Qingdao University, Qingdao, 266011 China

## Correction to: Stem Cell Research & Therapy (2022) 13:299 https://doi.org/10.1186/s13287-022-02978-x

Following the publication of this article, the authors regretfully found two errors in the article and would like to make corrections:

1. In the Methods-Histology and immunostaining section, “anti-PSA antibody (1:100, 10679-1-AP, Proteintech, China)” should be corrected to “anti-PSA antibody (1:100, AF0246, Affinity Biosciences, China; 1:100, 10679-1-AP, Proteintech, China)”. Both antibodies worked well in immunofluorescence and western blot experiments and rendered consistent results. In this paper, AF0246 was used for Figs. 2F and 4C, and 10679-1-AP for Figs. 5D and 7.
2. The word “PSA” mentioned in the article should be changed to “PSA homologous KLK protein” (or “KLK*” for short in the figures) and annotated “*mouse KLK homologous protein recognized by the PSA/KLK3 antibody” upon the first occurrence. We noticed that PSA (prostate-specific antigen), also called KLK3 (kallikrein related peptidase 3), is a human-specific gene, and mice express other KLK homologs. Blast results of the immunogens suggested that the PSA antibodies used in this paper might bind to other KLK family members in the mouse. In this paper, we used the PSA/KLK3 antibodies to label prostate luminal cells, as PSA is a marker of human prostate luminal cells. To verify the antibody specificity, we performed immunostaining on mouse prostate tissue cryosection and found that the antibody indeed labeled the luminal cells in the mouse prostate tissue (Additional file 1: Fig. S1), supporting the use of these antibodies in the paper.

Specifically, we want to make the following changes:

1. In the Abstract, change “PSA” to “PSA homologous KLK protein”.
2. Figures 2, 4, 5 and 7: in the figures, change “PSA” to “KLK*”; in the figure captions, change “PSA” to “KLK* (*mouse KLK homologous protein recognized by the PSA/KLK3 antibody)”.
3. Page 3, right column, “Histology and immunostaining”, lines 14–15, change “anti-PSA antibody (1:100, 10679-1-AP, Proteintech, China)” to “anti-PSA antibody (1:100, AF0246, Affinity, China; 1:100, 10679-1-AP, Proteintech, China)”
4. Change “PSA” to “PSA homologous KLK protein *” in the following sentences:
  Page 3, left column, lines 4 and 8
  Page 5, right column, line 5
  Page 7, left column, line 1,
  Page 8, right column, line 1
  Page 9, left column, line 1, right column, line 10
  Page 10, left column, line 3
  Page 11, right column, line 2 from bottom
  Page 12, left column, line 8

The corrected figures:

Figures 2, 4, 5 and 7.

**Fig. 2:**
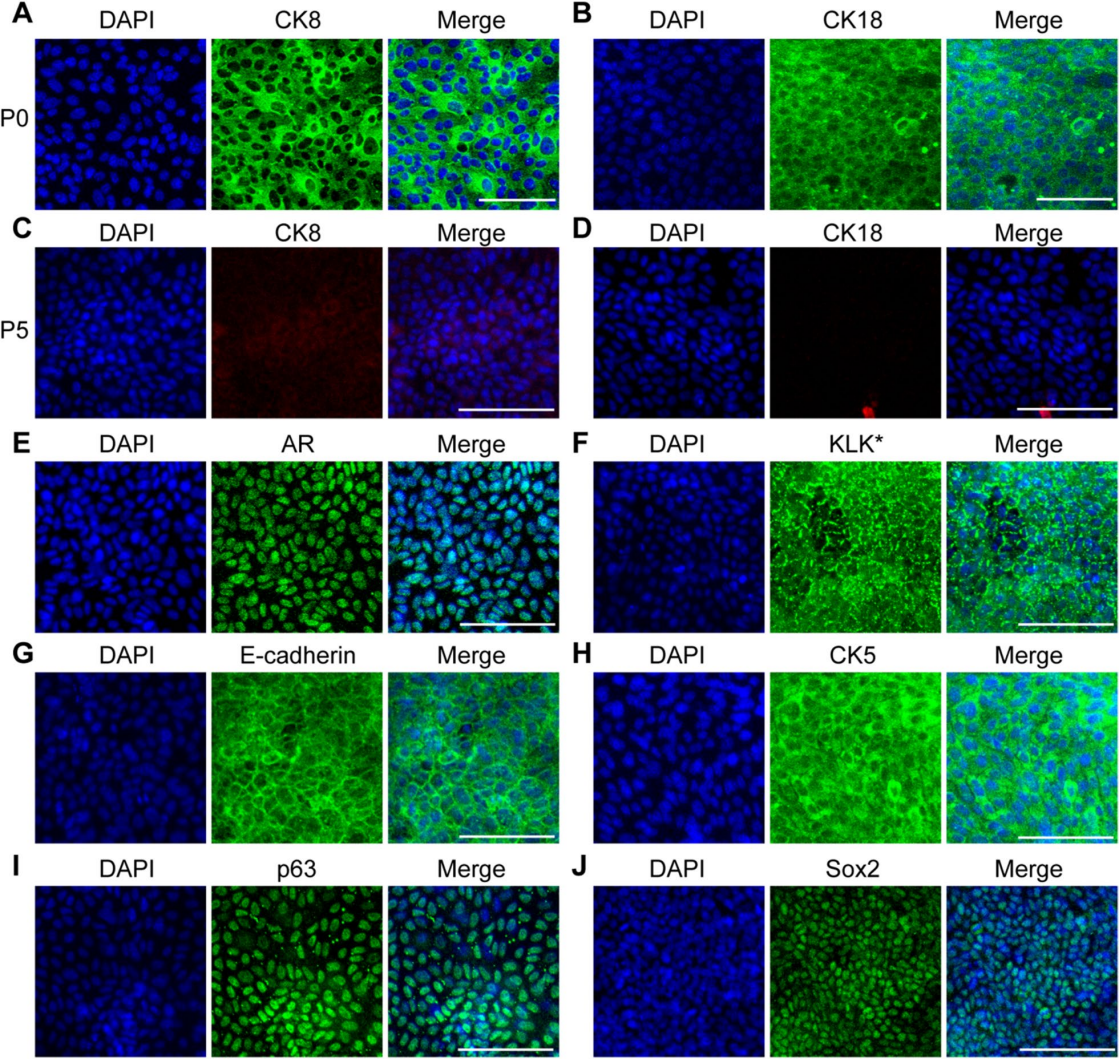
Marker expression of mouse CRIPSCs. The prostate epithelial cells isolated from WT mice at P0 (7 days) (**A** and **B**) and P5 (2 months) (**C**–**J**) were immunostained by the antibodies against CK8 (**A** and **C**), CK18 (**B** and **D**), AR (**E**), KLK* (*mouse KLK homologous protein recognized by the PSA/KLK3 antibody) (**F**), E-cadherin (**G**), CK5 (**H**), p63 (**I**), and Sox2 (**J**). DAPI-stained nuclei. Scale bars, 100 μm

**Fig. 4:**
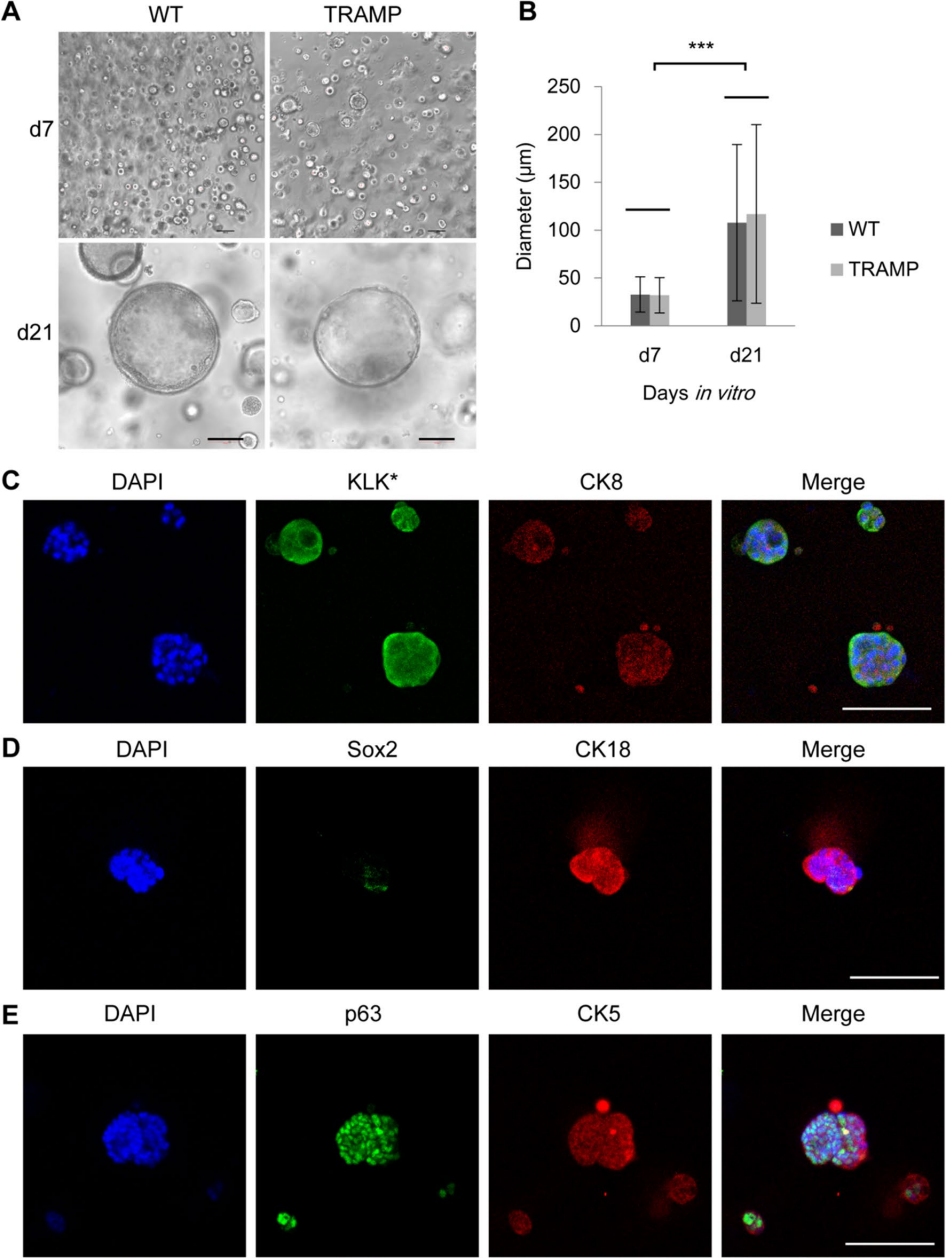
Organoid formation in vitro. **A** Phase-contrast images of the organoids formed by the CRIPSCs (P25, 6 months) derived from WT and TRAMP mice. **B** Quantification of organoid diameter. Data were presented as mean ± SD. Two-way ANOVA was performed on the data, followed by Bonferroni post hoc tests. ***p < 0.001. **C**–**E**, Immunofluorescence images of the organoids stained by the antibodies against CK8, CK18, KLK* (*mouse KLK homologous protein recognized by the PSA/KLK3 antibody), p63, CK5, and Sox2. DAPI-stained nuclei. Scale bars, 100 μm

**Fig. 5:**
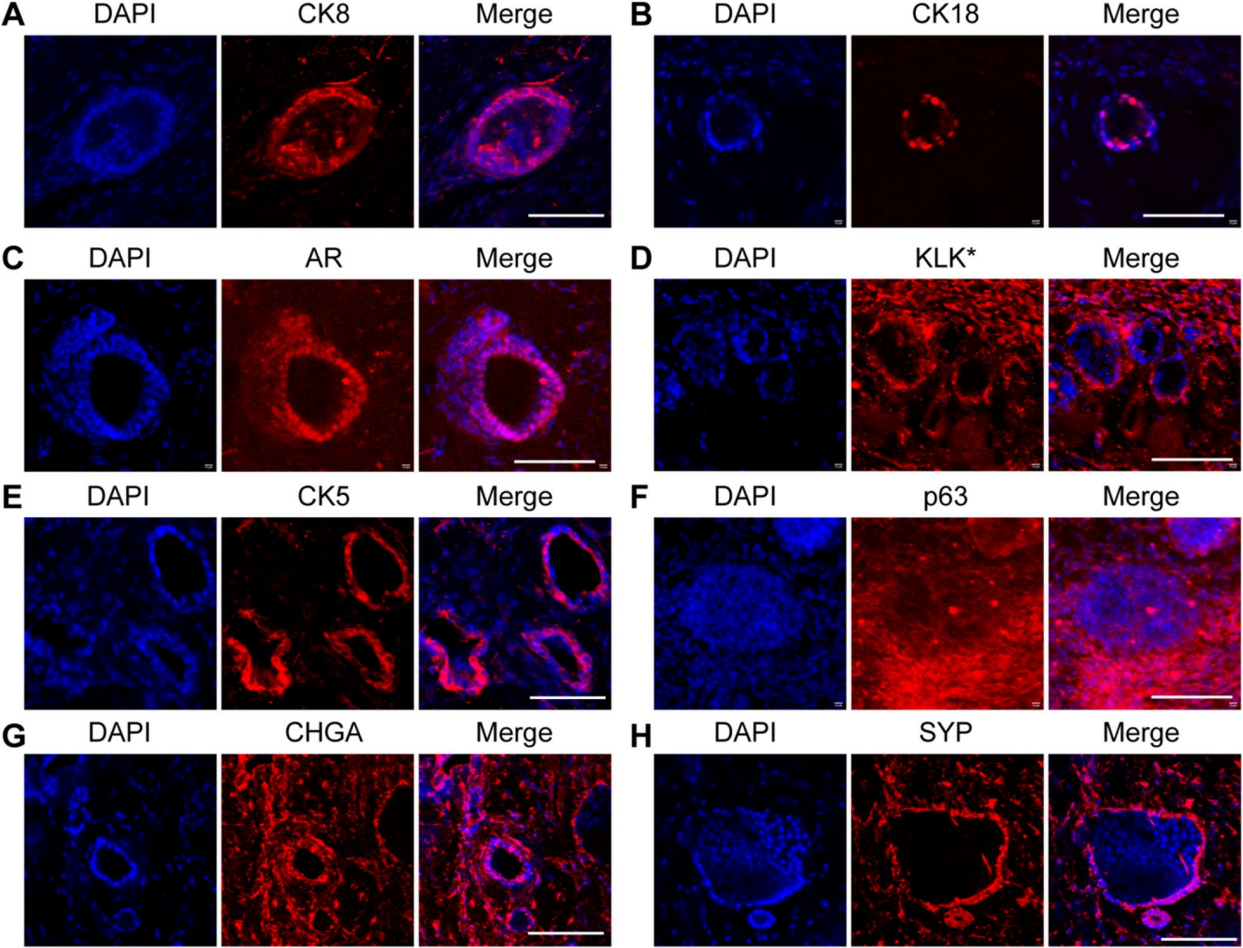
Differentiation of mouse CRIPSCs in vivo. The CRIPSCs (P25, 6 months) isolated from TRAMP mice were transplanted into NOG mice for eight weeks, followed by cryosection and immunostaining (**A**–**H**). The antibodies included CK8 (**A**), CK18 (**B**), AR (**C**), KLK* (*mouse KLK homologous protein recognized by the PSA/KLK3 antibody) (**D**), CK5 (**E**), p63 (**F**), Chromogranin A (CHGA) (**G**), Synaptophysin (SYP) (**H**). DAPI-stained nuclei. Scale bars, 100 μm

**Fig. 7:**
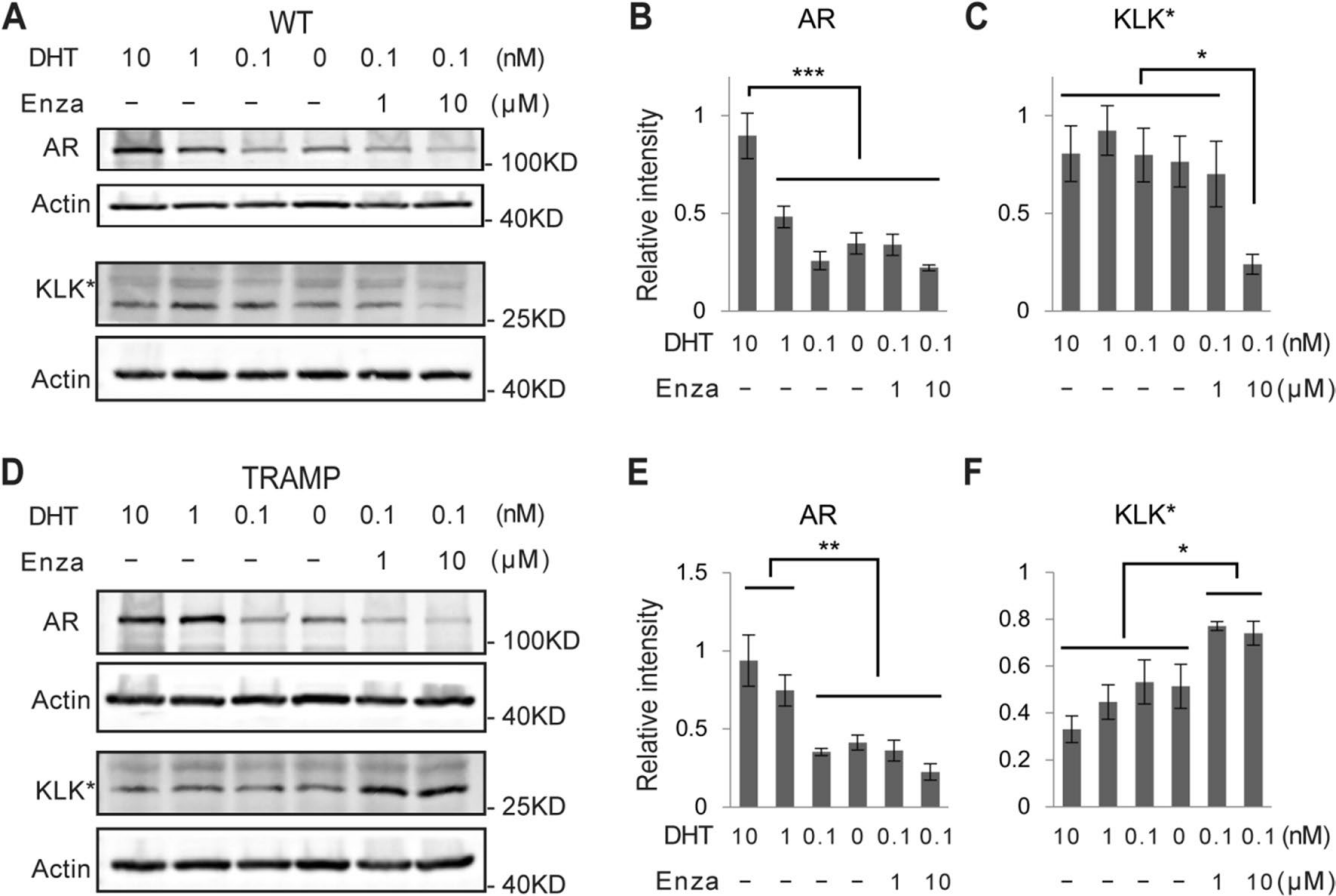
AR and KLK* (*mouse KLK homologous protein recognized by the PSA/KLK3 antibody) regulation by androgen deprivation and enzalutamide treatment. Western blots and quantifications of AR and KLK* of WT (**A**–**C**) and TRAMP (**D**–**F**) CRIPSCs (P25, 6 months) treated with different concentrations of DHT and enzalutamide (Enza) for one month. Data were presented as mean ± SD. One-way ANOVA was performed on the data, followed by Bonferroni post hoc tests. *p < 0.05. **p < 0.01. ***p < 0.001

These corrections will not affect the result and conclusion of the article. We sincerely apologize for any inconvenience caused.

## Supplementary Information

**Additional file 1.** **Supplementary Fig. 1.** Immunostaining of the prostate tissue cryosection of wild-type C57BL/6J mice. The antibody was against PSA/KLK3 from Affinity (Cat#AF0246). DAPI stained nuclei. Scale bars, 100 μm.

