# Supplementary material for: Correction: Expansion of mouse castration-resistant intermediate prostate stem cells in vitro
Source: Stem Cell Res Ther. 2022 Nov 25;13:522. doi: 10.1186/s13287-022-03207-1 (PMC9694920; doi:10.1186/s13287-022-03207-1)
Supplement: Supplementary file 1 — Additional file 1. Supplementary Fig. 1. Immunostaining of the prostate tissue cryosection of wild-type C57BL/6J mice. The antibody was against PSA/KLK3 from Affinity (Cat#AF0246). DAPI stained nuclei. Scale bars, 100 μm. [file 13287_2022_3207_MOESM1_ESM.docx]

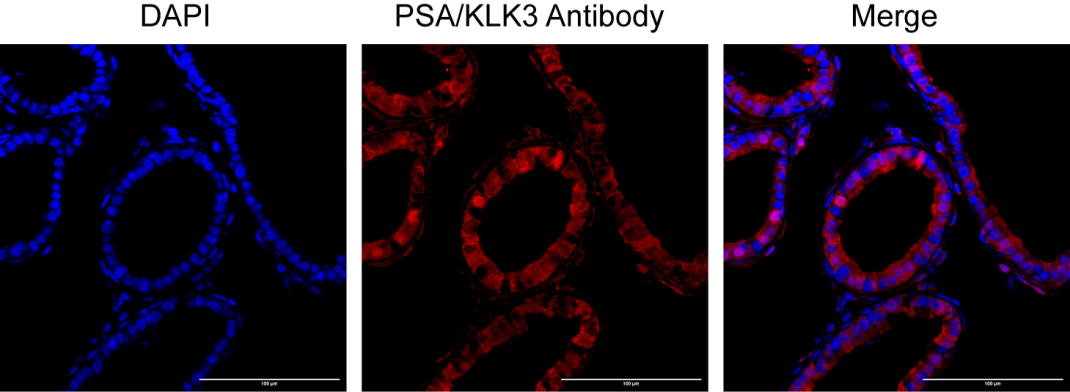


Supplementary Fig. 1. Immunostaining of the prostate tissue cryosection of wild-type C57BL/6J mice. The antibody was against PSA/KLK3 from Affinity (Cat#AF0246). DAPI stained nuclei. Scale bars, 100 μm.
